# Opposing Roles for Membrane Bound and Soluble Fas Ligand in Glaucoma-Associated Retinal Ganglion Cell Death

**DOI:** 10.1371/journal.pone.0017659

**Published:** 2011-03-29

**Authors:** Meredith S. Gregory, Caroline G. Hackett, Emma F. Abernathy, Karen S. Lee, Rebecca R. Saff, Andreas M. Hohlbaum, Krishna-sulayman L. Moody, Maura W. Hobson, Alexander Jones, Paraskevi Kolovou, Saoussen Karray, Andrea Giani, Simon W. M. John, Dong Feng Chen, Ann Marshak-Rothstein, Bruce R. Ksander

**Affiliations:** 1 The Schepens Eye Research Institute, Department of Ophthalmology, Harvard Medical School, Boston, Massachusetts, United States of America; 2 Department of Microbiology, Boston University School of Medicine, Boston, Massachusetts, United States of America; 3 Department of Medicine, Harvard Medical School, Boston, Massachusetts, United States of America; 4 Department of Medicine, University of Massachusetts Medical School, Worcester, Massachusetts, United States of America; 5 Institut National de la Sante et de la Recherche Medicale (INSERM) Unite 580, Hopital Necker, Paris, France; 6 Massachusetts Eye and Ear Infirmary, Department of Ophthalmology, Harvard Medical School, Boston, Massachusetts, United States of America; 7 Howard Hughes Medical Institute, Jackson Laboratory, Bar Harbor, Maine, United States of America; Alcon Research, Ltd., United States of America

## Abstract

Glaucoma, the most frequent optic neuropathy, is a leading cause of blindness worldwide. Death of retinal ganglion cells (RGCs) occurs in all forms of glaucoma and accounts for the loss of vision, however the molecular mechanisms that cause RGC loss remain unclear. The pro-apoptotic molecule, Fas ligand, is a transmembrane protein that can be cleaved from the cell surface by metalloproteinases to release a soluble protein with antagonistic activity. Previous studies documented that constitutive ocular expression of FasL maintained immune privilege and prevented neoangeogenesis. We now show that FasL also plays a major role in retinal neurotoxicity. Importantly, in both TNFα triggered RGC death and a spontaneous model of glaucoma, gene-targeted mice that express only full-length FasL exhibit accelerated RGC death. By contrast, FasL-deficiency, or administration of soluble FasL, protected RGCs from cell death. These data identify membrane-bound FasL as a critical effector molecule and potential therapeutic target in glaucoma.

## Introduction

Glaucoma is one of the most common causes of blindness worldwide and, while there are many different forms of glaucoma that differ significantly in clinical presentation and disease progression, they all share a common endpoint which is the loss of retinal ganglion cells (RGCs) [1]. One of the most common forms of glaucoma, primary open angle glaucoma, is associated with increased intraocular pressure. However, patients with low or normal intraocular pressure can also develop glaucoma, indicating that mechanisms independent of elevated pressure contribute to the death of RGCs. In spite of extensive research, the pathobiology of glaucoma is poorly understood. Recent evidence indicates the loss of RGCs is due to apoptosis [2], nevertheless, the actual molecular mechanism that triggers apoptosis is controversial.

Data from clinical studies and animal models of induced elevated intraocular pressure (IOP) support the hypothesis that there is an inflammatory component to glaucoma and that TNFα contributes to disease progression. Elevated levels of TNFα have been detected in the aqueous humor and retinal layers of glaucoma patients with primary open angle, normal tension, and exfoliation glaucoma [3] [4]. In addition, TNFα polymorphisms have been associated with primary open angle glaucoma in Japanese and Chinese populations [5], [6]. Development of glaucoma also coincided with increased levels of TNFα and TNFα-inducible genes in laser induced rodent models of elevated IOP [7] [8]. However, in this model, TNFα did not appear to directly induce cytolysis of RGCs, although it could be shown to activate microglia [7]. Moreover, a single injection of TNFα into the vitreous of eyes with normal pressure triggered the loss of RGCs. Together, these data indicate that ocular stress, such as elevated intraocular pressure, can trigger the release of TNFα, which in turn activates microglia to become neurotoxic for RGCs. However, the direct effector mechanism responsible for microglia mediated RGC neurotoxicity is not TNFα.

Fas Ligand (FasL) is one candidate that may link activation of microglia with the induction of apoptosis in RGCs. Fas Ligand (FasL) is a 40 kDa type II transmembrane protein of the TNF family, originally identified by its capacity to induce apoptosis in Fas receptor positive cells [9] and mediate activation induced cell death in T cells [10]. FasL is expressed by activated T cells and constitutively expressed on ocular tissues where it is thought to contribute to the immune privileged status of the eye, either by inducing apoptosis of infiltrating inflammatory cells or by preventing neoangeogenesis [11]. In addition to its pro-apoptotic activity, FasL can also induce the release of proinflammatory cytokines [12], [13], [14]. Importantly, in a rat model of heat-shock protein-induced RGC degeneration, FasL+ autoreactive T cells have been implicated in the damage of Fas+ RGCs [15]. By contrast, RGC degeneration in the laser-induced ocular hypertension models does not appear to involve T cells. However, other cells of the innate immune system, notably macrophages and retinal microglia, can express FasL upon activation [16], [17]. Thus FasL+ effector cells could be involved in T-independent destruction of RGCs. Such a pathogenic role for FasL appears to be in conflict with its purported role in immune privilege.

The diverse activities of FasL could result from functional differences in cell-bound vs soluble forms of the molecule, as is true for other TNF family members [18], [19], [20]. Moreover, FasL can be released from the cell by at least two mechanisms. FasL can be cleaved from the cell surface by metalloproteinases to produce a truncated soluble product derived from the extracellular domain (sFasL) [21]. In addition, cell lines and activated T cells have been reported to release full-length FasL in the form of microvesicles [22], [23]. Both truncated sFasL and full-length vesicle-associated FasL can be detected as cell-free FasL by standard ELISA readouts, leading to some confusion as to the valency and functional activity of cell-free FasL. There is considerable data to suggest that murine sFasL is non-apoptotic and anti-inflammatory, and in some instances, sFasL has even been shown to antagonize the activity of mFasL. This is in contrast to an experimental form of FasL that corresponds to the entire extracellular domain [24], [25]. On the other hand, sFasL bound to extracellular matrix proteins is cytotoxic and FasL has been localized to the extracellular matrix in the anterior chamber of the eye [26]. Thus, whether FasL accumulates in the ocular environment as full-length mFasL or truncated sFasL, matrix-associated or not, could influence its functional consequences. Remarkably, the relative levels of full-length and cleaved FasL in the eye have not been carefully evaluated.

Most direct functional comparisons of mFasL and sFasL have been carried out by using transfected cells that express only wild type FasL, mFasL or sFasL. In the current study, we have used FasL-deficient mice as well as mice from a gene-targeted line in which the FasL metalloproteinase cleavage sites were mutated to prevent cleavage of the membrane-bound protein. We have compared the ability of these mice to develop RGC degeneration following intraocular TNFα treatment and in a spontaneous model of glaucoma. Overall our data reveal a critical neurotoxic effector function for mFasL and neuroprotective function of sFasL.

## Results

### TNFα triggered loss of RGCs is dependent upon FasL

**Figure 1:**
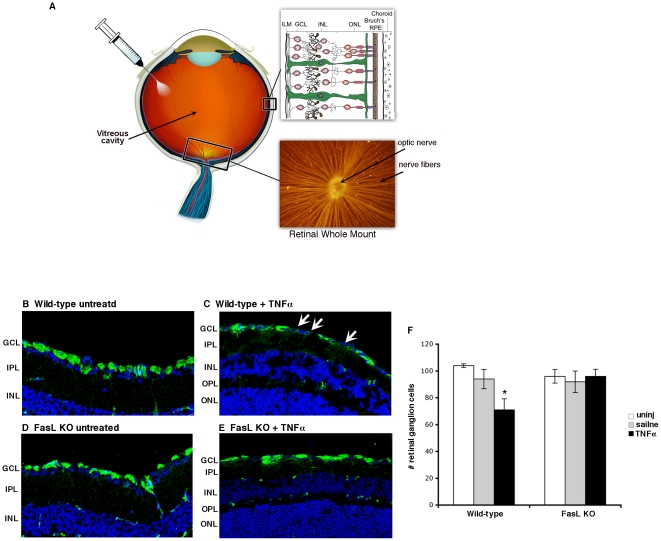
FasL is required for the loss of RGCs following TNFα injection. (A) Diagram of the cross section of an eye demonstrating: an intravitreal TNFα injection; the layers of the retina (ILM- inner limiting membrane, GCL- ganglion cell layer, INL- inner nuclear layer, ONL- outer nuclear layer); a retinal whole mount stained to identify the nerve fiber layer. (B–E) WT C57BL/6 or FasL KO mice were untreated or received intravitreal injections of saline or TNFα. Eyes were enucleated at 4 weeks and RGCs were identified in retinal sections using an anti-βIII-tubulin antibody (green) and TOPRO (blue nuclear stain). (F) All βIII-tubulin positive RGCs were counted in each retinal section and the average number RGCs per retinal section was calculated (10 sections per eye). N = 10 per treatment group. (* P<0.05).

To examine the role of Fas/FasL interactions in the death of RGCs, we used the intravitreal TNFα injection model developed by Nakazawa et al (see **Figure 1A**) [7]. Recipients included a FasL knockout (KO) line, developed in our laboratory (**Figure S1**). As the more commonly used point mutation encoded by the FasL*^gld^* locus does not completely eliminate Fas receptor engagement [27]. Groups of wild-type C57BL/6J (WT-B6) and FasL KO mice were given a single intravitreal injection of TNFα (1 ng/0.5 μl). As negative controls, mice were either untreated, or injected with normal saline. At four weeks post injection, retinal sections were stained with an anti-β-III-tubulin antibody to identify RGCs (**Figure 1 B–E**). The number of RGCs was determined quantitatively in representative retinal sections as described in the methods (**Figure 1F**). There was no significant difference in the number of RGCs between either untreated WT, WT mice given an intravitreal injection of saline, or untreated FasL KO mice. Therefore the absence of FasL did not have any spontaneous effect on the number of RGCs. However, consistent with previous studies, a single intravitreal injection of TNFα resulted in a significant loss of RGCs in the WT-B6 mice by four weeks post TNFα injection. By contrast, there was no loss of RGCs in TNFα treated FasL KO mice. We conclude from these experiments that FasL is required for the TNFα triggered loss of RGCs.

### ΔCS mice express only mFasL and maintain normal ocular histology

**Figure 2:**
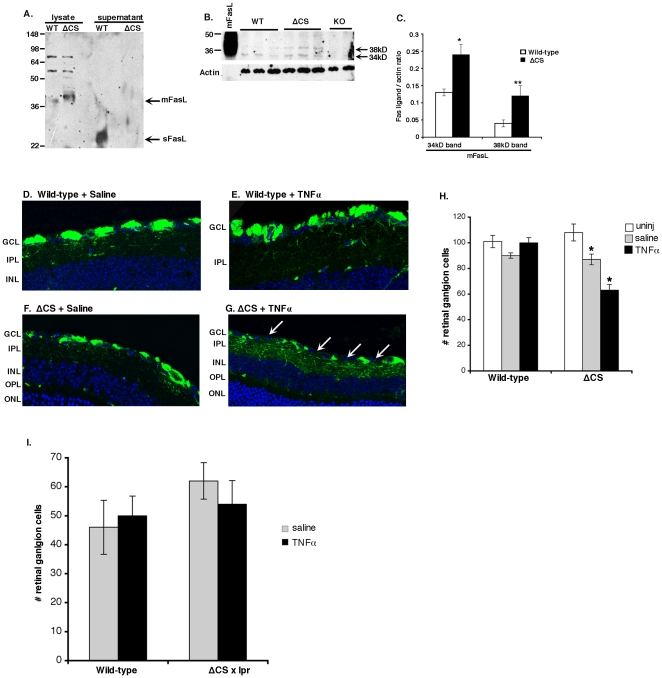
Loss of RGCs in ΔCS mice is dependent upon the Fas/FasL pathway. All Western blots displayed are representative of three independent experiments: (A) CD3 activated T cell lysates and supernatants, (B) whole eye lysates from individual eyes of: WT, ΔCS, and FasL KO mice, (C) densitometry of eye lysate Western blots (* and ** P<0.05), (D–G) WT or ΔCS mice received an intravitreal injection of saline, or TNFα. Retinal sections were obtained 7 days later and RGCs were stained with βIII-tubulin. Arrow heads highlight the loss of RGCs. (H) All βIII-tubulin positive RGCs were counted in each retinal section (5 sections per eye; 10 eyes per group) and the average number RGCs per retinal section was calculated. (I) WT or ΔCS×lpr mice received intravitreal injections of saline, or TNFα. The number of RGCs was determined 7 days later in retinal sections. Green = Beta tubulin III and blue = nuclear stain. N = 10 per treatment group. (* p>0.05).

To examine the importance of sFasL in ocular homeostasis and RGC degeneration, we constructed a membrane-only FasL gene-targeted mouse in which the FasL metalloproteinase cleavage sites in exon 2 were mutated (**Figure S1**). This mouse line was designated as ΔCS. WT and ΔCS mice were tested for expression of mFasL and sFasL. Cell lysates and culture supernatants prepared from activated T cells were analyzed by Western blot. A 38 kDa mFasL band was detected in the WT lysate and a 27 kDa sFasL band was detected in the WT supernatant. By contrast, cell lysates from the ΔCS T cells contained more of the 38 kDa mFasL protein and the ΔCS supernatant contained no detectable sFasL (**Figure 2A**). Whole eye lysates were also prepared from WT and ΔCS mutant mice to test for ocular expression of mFasL and sFasL by Western blot (**Figure 2B**). Two mFasL bands (38 kD and 34 kD) were detected at low levels in whole eye lysates from WT mice. As an important specificity control, both mFasL bands were missing from eye lysates that were prepared from FasL knockout mice [27]. Therefore, the two bands most likely represent differential glycosylation, previously reported for mFasL [24], [28], [29]. In comparison to WT mice the expression of both the 38 kD and 34 kD mFasL bands were significantly increased in ΔCS mice (**Figure 2B and 2C**). It is important to note, that sFasL was not detected by Western blot in the whole lysates from WT mice, possibly reflecting clearance from the eye or instability of the soluble form.

Altogether, these data demonstrate that ΔCS mutant mice express increased levels of mFasL and no detectable sFasL. Importantly, the ΔCS mutant mice display no clinically detectable spontaneous systemic phenotype. Extensive histological studies of ocular tissues did not reveal any detectable spontaneous eye phenotype in either the anterior or posterior segment.

### Accelerated loss of RGCs in ΔCS mice

Groups of WT and ΔCS mice received a single intravitreal injection of TNFα (1 ng/0.5 μl) and 1 week later mice were euthanized and the eyes evaluated histologically for RGC degeneration (**Figure 2 D–H**). Untreated ΔCS and WT mice displayed no significant difference in the number of RGCs, indicating the ΔCS mutation had no visible effect on RGC development in healthy mice. As expected from the previous work of Nakazawa [7], WT mice treated with TNFα did not display any significant loss of RGCs 1 week after administration; normally a reduction of RGCs in WT mice is not observed until 4 weeks after TNFα treatment. By contrast, there was a significant reduction in the number of RGCs in ΔCS mice treated with TNFα. Moreover, there was a small, but significant reduction in the number of RGCs in ΔCS mice that received an intravitreal saline injection. In this case, RGC loss is most likely due to a hypersensitive response to the modest release of TNFα that is triggered by the injection procedure. These data indicate that the increased expression of mFasL by microglia (or other effector cells), and/or the absence of sFasL in the ocular microenvironment, results in an accelerated loss of RGCs in response to TNFα.

To prove the ΔCS mutation accounted for the accelerated loss of RGCs via the Fas receptor, we intercrossed ΔCS mice with Fas-deficient lpr mice. B6/129^ΔCS/ΔCS lpr/lpr^ and wild type littermates expressing normal FasL and Fas were given intravitreal injections of either TNFα or normal saline. One week post TNFα injection, RGC degeneration was evaluated histologically. In Fas-deficient ΔCS mice, TNFα was unable to trigger the loss of RGCs (**Figure 2I**). Together these data prove that the accelerated loss of RGCs in TNFα injected ΔCS mice is dependent upon the Fas/FasL pathway.

### Loss of nerve fibers in the retina of ΔCS mice

**Figure 3:**
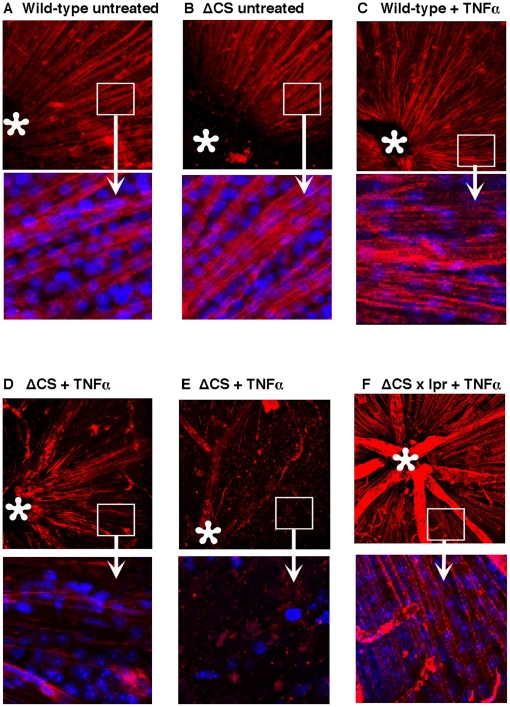
The loss of retinal ganglion cell nerve fibers is accelerated in ΔCS mice. WT, ΔCS, or ΔCS×lpr mice were either untreated, or received intravitreal TNFα. Seven days later the nerve fibers in retinal whole mounts were stained with SMi32 (anti-neurofilament antibody) and examined by confocal microscopy. (A) untreated WT retina, (B) untreated ΔCS retina, (C) WT+TNFα treated retina, (D and E) ΔCS+TNFα treated retina, (F) ΔCS×lpr+TNFα treated retina. Asterisks mark the optic nerve head, Red = SMi32, and blue = nuclear stain. The pictures presented are representative of individual mice (N = 10 for each group).

Glaucoma is characterized not only by the loss of RGCs, but also by the loss of the their axons. The axonal loss is often visualized clinically as slit-like or wedge-shape defects in the retinal nerve fiber layer. To evaluate the effect of FasL triggered RGC death on axonal integrity, we examined the nerve fiber layer of WT and ΔCS mice in retinal whole mounts (**see diagram in** **Figure 1A**). There was no significant difference between the nerve fiber layers of WT and ΔCS mice that were either uninjected (**Figure 3 A, B**), or administered an intravitreal injection of saline (data not shown). Moreover, WT mice treated with TNFα displayed a normal nerve fiber layer one week after treatment that was not significantly different from the untreated controls (**Figure 3C**). By contrast, at 1 week after treatment, the TNFα-treated ΔCS mice displayed a significant loss of nerve fibers (**Figure 3D**), with some mice displaying very few intact axons (**Figure 3E**). Axonal loss in the TNFα treated ΔCS mice was abrogated in B6/129^ΔCS/ΔCS lpr/lpr^ mice that lacked a functional Fas receptor (**Figure 3F**). These data demonstrate that ΔCS mice exhibit accelerated loss of both the soma and axon in response to TNFα.

### Exogenous sFasL prevents the accelerated loss of RGC in ΔCS mice

**Figure 4:**
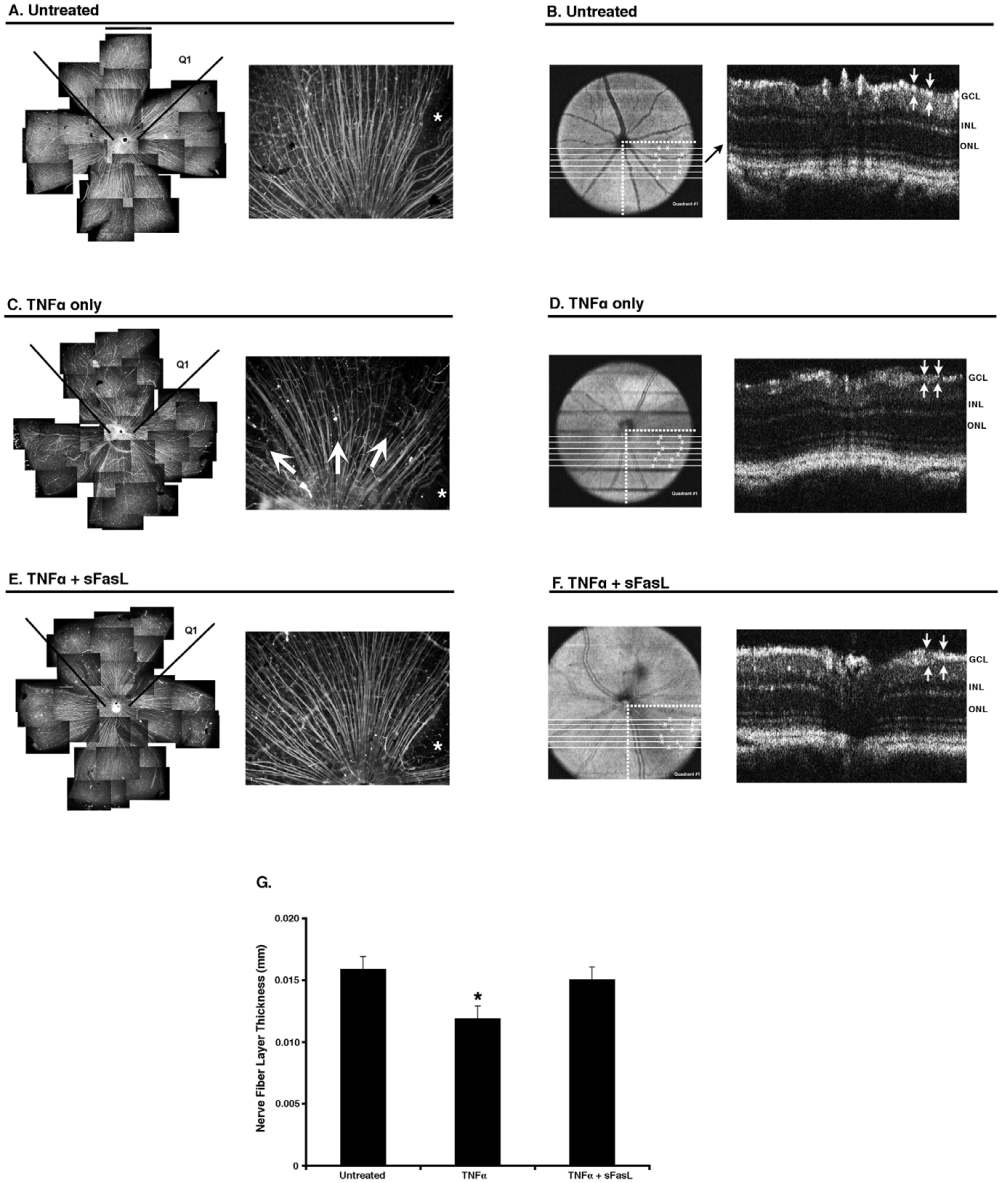
sFasL protects retinal ganglion cell nerve fibers in ΔCS mice. ΔCS mice were either untreated, or received intravitreal: TNFα (1 ng) alone, or TNFα+sFasL. Seven days later SD-OCT measurements were made and subsequently the nerve fibers in retinal whole mounts were stained with Smi32 (anti-neurofilament antibody). Displayed are a low power composite montage photograph of the entire Smi32 stained retina and a higher power (×20 magnification) of quadrant #1 (Q1). (A) untreated, (C) TNFα alone, and (E) TNFα+sFasL. Asterisks (*) indicate areas where retinas were dissected. Arrows highlight nerve fiber thinning. Pictures are representative of a single mouse from each group (N = 10 per group). The quadrant #1 was identified in the OCT en-face fundus reconstruction in (B) untreated, and (D) TNFα alone, and (F) TNFα+sFasL. Six sections within the quadrant were chosen and the nerve fiber thickness measured at two points in each section (identified by an X). A single OCT retinal section and the corresponding nerve fiber measurements are displayed. (G) A summary of the OCT measurements for each group (N = 3 per group; two experiments performed). (* p>0.05).

We and others demonstrated previously that mFasL is proinflammatory and proapoptotic, while sFasL is anti-inflammatory and non-apoptotic [24]. To determine whether administration of recombinant sFasL could prevent the loss of RGCs in TNFα treated ΔCS mice, ΔCS mice received an intravitreal injection of either sFasL alone (100 ng), TNFα alone (1 ng), or both sFasL and TNFα. At 7 days post injection, the eyes were evaluated for axonal degeneration in retinal whole mounts. No significant loss of nerve fibers was observed in either WT, or ΔCS mice treated with sFasL alone as compared with untreated WT and ΔCS mice (data not shown). As expected, a significant loss in nerve fibers was observed at 7 days post TNFα treatment in ΔCS mice compared to the untreated control mice (**Figure 4A, 4C**). However, intravitreal injection of recombinant soluble Fas ligand together with TNFα prevented the loss of nerve fibers (**Figure 4E**). It is important to note that the mouse recombinant sFasL used in these experiments corresponds with the physiological cleavage product (Pro 132 to Leu 279) and has weak or no cytolytic activity against A20 target cells.

Spectral domain optical coherence tomography (SD-OCT) provides a noninvasive method to assess the thickness of the retinal nerve fiber layer [30]. In the clinic, the retinal nerve fiber layer thickness is an essential measure for objective glaucoma assessment [31], [32]. However, the use of SD-OCT to measure the retinal nerve fiber layer thickness in the mouse is a relatively new area of investigation. In the current study, SD-OCT was used to assess the retinal nerve fiber layer thickness prior to enucleation and preparation of retinal whole mounts. Sectorial areas containing retinal defects were identified in retinal whole mounts stained with SMI32 (**quadrant #1 in** **Figure 4A, 4C, and 4E**). The corresponding retinal areas were identified in the OCT-based en-face fundus reconstruction and nerve fiber thickness was assessed as described in the methods (**Figure 4B, 4D, and 4F**). SD-OCT measurements indicated a significant thinning of the nerve fiber layer in TNFα treated, but not TNFα+sFasL treated mice (**Figure 4G**). These data indicate that sFasL can block the neurotoxic effects of membrane FasL and prevent nerve fiber loss in ΔCS mice. Moreover, these date indicate that SD-OCT can be used as a non-invasive method to assess retinal nerve fiber layer thickness in mice.

### Retinal microglia express mFasL

**Figure 5:**
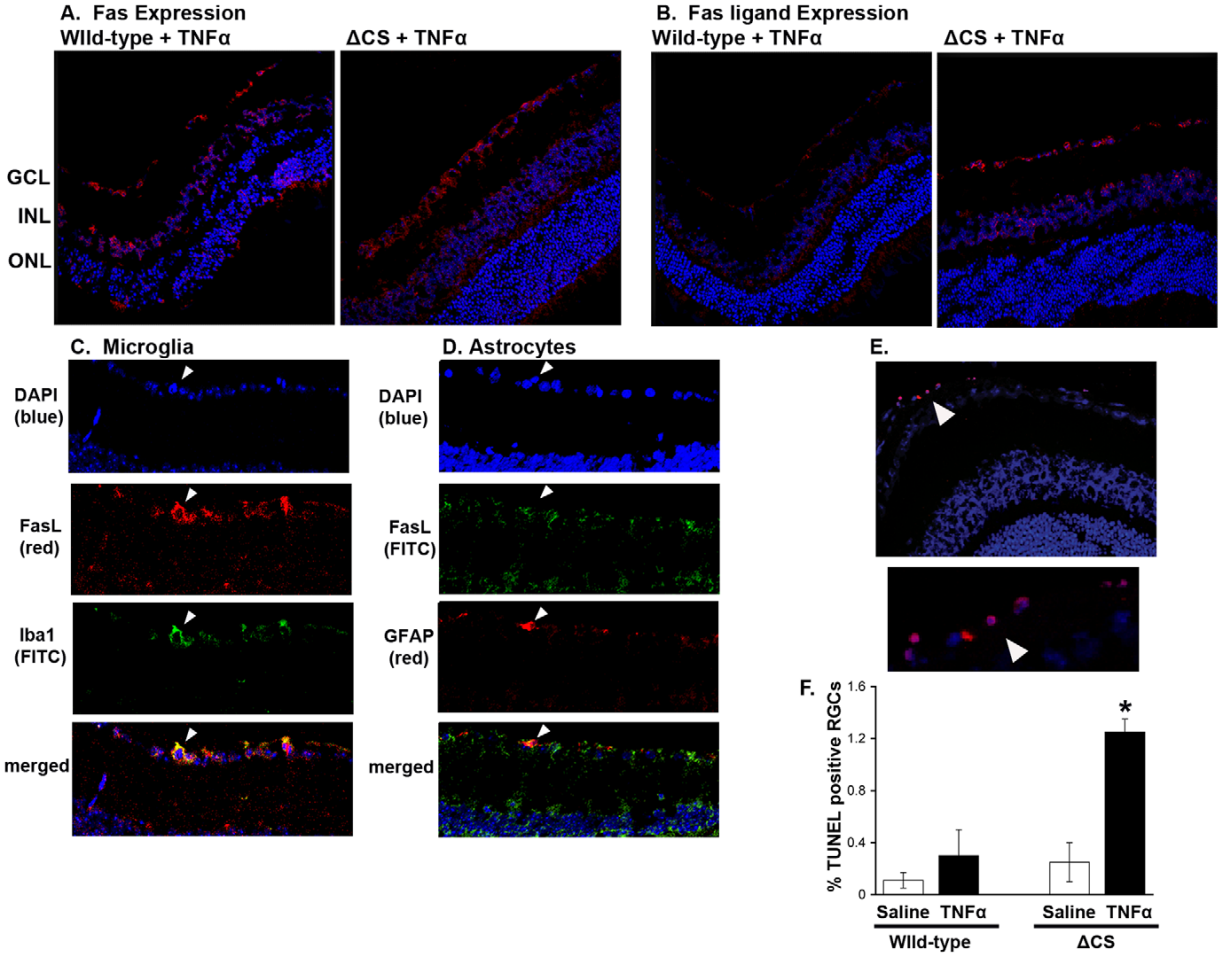
Expression of Fas and FasL in the neural retina of WT and ΔCS mice. Frozen retinal sections were prepared from WT and ΔCS mice that were either (i) untreated, or (ii) received a prior (7 days) intravitreal injection of TNFα. (A) Fas receptor expression using an anti Fas antibody (Red) and TOPRO (blue nuclear stain). (B) FasL expression using an anti FasL antibody (red) and TOPRO (blue nuclear stain). Identically treated retinal sections from FasL KO mice were used as a negative control. (C) Double staining for microglia (Iba1-green) and FasL (red) revealed retinal microglia (arrowhead) express FasL. (D) Double staining for astrocytes (GFAP-red) and FasL (green) revealed retinal astrocytes (arrowhead) were FasL negative. (E) Representative TUNEL staining in ΔCS mice at 24 hours post TNFα injection. Red = TUNEL, Blue = nuclear stain. GCL- ganglion cell layer; INL- inner nuclear layer; ONL- outer nuclear layer. (F) Percentages of TUNEL positive cells in the retina. (N = 5 per group). (* p>0.05). See also Figure S2.

Retinal tissue from TNFα treated WT and ΔCS mice were examined by immuno-histochemical staining for Fas and FasL to confirm that Fas+ target cells were present within the ganglion cell layer and to determine which cell types were potential FasL effector cells. As expected, Fas was highly expressed in the ganglion cell layer and the level of Fas was similar in TNFα treated WT and ΔCS mice (**Figure 5A**).

In contrast to Fas, FasL was expressed at much higher levels in ΔCS mice as compared to WT mice. Moreover, the most intense staining was observed in the ganglion cell layer, with some staining also observed in the inner nuclear layer (**Figure 5B**). Double staining for microglia (Iba1) and astrocytes (GFAP) clearly demonstrated that FasL expression in the ganglion cell layer was primarily restricted to retinal microglia (**Figure 5C**) and minimally, if at all, in astrocytes (**Figure 5D**). These data indicate that ΔCS retinal microglia express higher than normal levels of mFasL and/or that greater numbers of FasL+ microglial cells accumulate in the TNF-treated ΔCS retinae [33].

### RGCs are susceptible to mFasL induced apoptosis

We verified that the Fas+ human retinal ganglion cell line, RGC5, was susceptible to mFasL induced apoptosis using mFasL expressing microvesicles (**Figure S2**). To determine whether TNFα treatment of ΔCS mice triggered apoptosis of RGCs in vivo, ΔCS and WT mice were treated with TNFα and at 24 hrs post injection histological sections were stained with TUNEL and examined for apoptotic cells. No apoptotic cells were detected in sections from untreated WT or untreated ΔCS mice (data not shown). In addition, little to no apoptosis was observed in saline treated WT and ΔCS mice. However, a significant number of apoptotic cells were detected in the retinal ganglion cell layer of TNFα treated ΔCS mice (**Figure 5 E, F**). Together these data support the hypothesis that TNFα activated retinal microglia express mFasL that triggers apoptosis of Fas receptor positive RGCs.

### mFasL induced retinal degeneration in a spontaneous model of glaucoma

**Figure 6:**
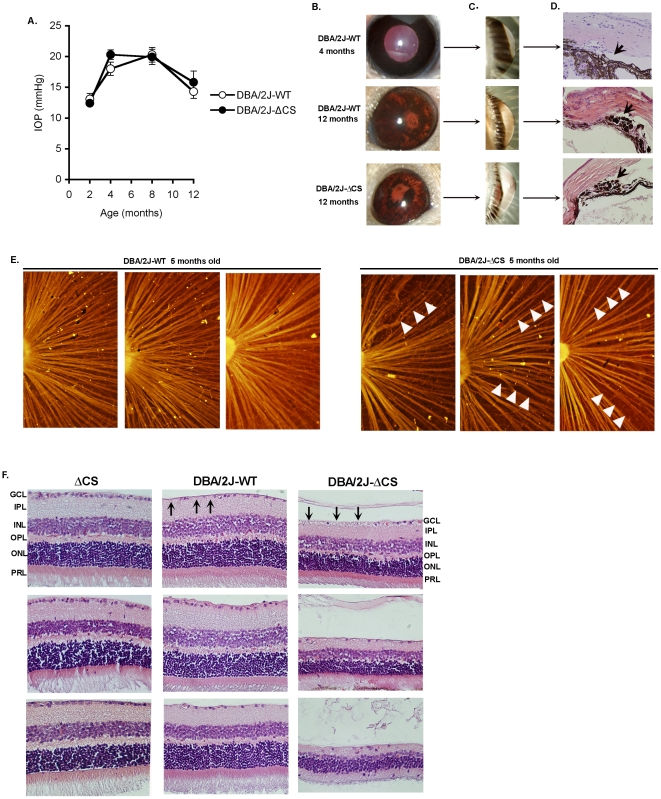
mFasL induced retinal degeneration in DBA/2J mice. The ΔCS mutation was backcrossed to DBA/2J mice (DBA/2J-ΔCS) and compared with littermate controls (DBA/2J-WT). (A) intraocular pressure (IOP) (N≥10 mice per group; mean +/− SEM). DBA/2J-WT and DBA/2J-ΔCS (12 mons old) were compared with young (4 mons old) DBA/2J mice for: (B) pigment dispersion and iris atrophy, (C) size of the anterior chamber, and (D) H&E sections of the iridocorneal angle revealing pigment laden cells (arrow) blocking the aqueous outflow pathway. (E) retinal whole mounts stained with Smi32 (anti-neurofilament antibody); arrows identify thinning of the nerve fibers. (F) H&E stained retinal sections from: B6×129 ΔCS mice (10 mons old), DBA/2J-WT (12 mons old), and DBA/2J-ΔCS (12 mons old). The pictures presented are representative of individual mice in each group (N = 10).

DBA/2 mice spontaneously develop age-related elevated intraocular pressure due to mutations in the *Gpnmb* and *Tyrp1* genes that trigger iris stromal atrophy and pigment dispersion, respectively. This results in closure of the iridocorneal angle and elevated IOP by approximately 6–8 months of age, followed by the loss of RGCs and nerve fibers between 11 and 15 months [34], [35]. To determine if mFasL also accelerates RGC degeneration in this spontaneous model of glaucoma, we backcrossed the ΔCS mutation onto the DBA/2J background. The 5th generation backcross mice were intercrossed to obtain ΔCS/ΔCS mice (DBA/2J- ΔCS), and a WT/WT littermate control group (DBA/2J-wt). Both the DBA/2J-wt and DBA/2J- ΔCS mice developed high intraocular pressure with age (**Figure 6A**), pigment dispersion (**Figure 6B**), an enlarged anterior chamber (**Figure 6C**), and angle closure (**Figure 6D**). The appearance of these symptoms was not significantly different from fully backcrossed DBA/2J mice as reported previously [34], [35]. Thus the failure to cleave FasL in DBA/2J- ΔCS mice did not ameliorate these glaucoma-inducing phenotypes.

However, while there was no detectable loss of nerve fibers in 5 month old DBA/2J-wt mice, there was marked thinning of the nerve fibers in the DBA/2J- ΔCS mice (**Figure 6E** arrows), indicative of accelerated loss of axons. Previous extensive analysis of 12 month old DBA/2J mice by Jakobs and coworkers indicated that elevated intraocular pressure coincided with loss of *only* RGCs; no other retinal neurons were affected [36]. This is evident from the loss of cells in the ganglion cell layer in 12 month old DBA/2J-wt mice (**Figure 6F**, arrows). Unexpectedly, when the DBA/2J- ΔCS mice reached 12 months of age, they displayed not only a greater loss of ganglion cells and nerve fibers, but also extensive retinal degeneration in all layers of the retina (**Figure 6F**). This was not due to the presence of the ΔCS mutation alone, since 10 month old ΔCS B6×129 mice displayed normal retinal architecture and no loss of RGCs. Together, these data indicate that in a spontaneous elevated intraocular pressure model of glaucoma, mFasL is also highly neurotoxic.

## Discussion

Fas ligand is a potent pro-apoptotic molecule expressed by cytotoxic effector T and NK cells that is known for its ability to eliminate virally infected target populations, tumor cells, and autoreactive T and B cells [11]. However, persistent expression of FasL by Fas-deficient T cells in mice with Fas+ non-T cell populations can result in graft-vs-host-like disease [37], total elimination of wild type lymphocytes [38], or can even cause pulmonary fibrosis [39]. In addition, FasL has also been shown to rapidly induce the production of proinflammatory cytokines by a variety of Fas+ cell types and to promote T lymphocyte activation [13]. Therefore it is not surprising that expression of FasL on T and NK cells is tightly regulated either at the transcriptional level [40], by sequestration in cytoplasmic vesicles [41], or by metalloprotease cleavage [13].

Remarkably, in contrast to T cells and NK cells, FasL is constitutively expressed at sites of immune privilege, such as the eye. Ocular expression of FasL is thought to be required for the maintenance of immune privilege by limiting ocular inflammation [42] and/or neoangeogenesis [43]. Consistent with this notion, FasL deficient mice develop a more severe inflammatory response in a murine model of acquired ocular taxoplasmosis [44]. Natural expression of ocular FasL (i) promotes engraftment of allogeneic corneal transplants by inducing apoptosis of infiltrating Fas+ activated T cells [42], and (ii) prevents suture induced neovascularization by inducing apoptosis of vascular endothelial cells [45]. By contrast, FasL-deficient mice present with increased corneal graft rejection and suture induced neovascularization [45], [46]. More recent studies, however, reveal an apparent paradox in FasL function within the ocular environment, where ocular expression of membrane-bound FasL actually promotes immunoreactivity. For example: (i) over expression of non-cleavable FasL in the cornea triggers accelerated transplant rejection [47], and (ii) tumor cells that express a membrane-only form of FasL induce a severe ocular inflammatory response [48] Moreover, a number of studies have shown that the soluble form of FasL antagonizes the functional outcome of membrane-bound FasL [24], [25], [49], while others reported that sFasL could bind to ocular matrix proteins and thereby acquire potent apoptotic activity [26]. Thus it is unclear how immune privileged sites regulate FasL activity and control its potentially dangerous effects related to inflammation and apoptosis of host tissues.

As previously documented [50] and confirmed in the current report, whether or not a mouse either fails to express FasL, or over expresses membrane-bound FasL, does not appear to affect ocular development or normal lymphocyte homeostasis. Since numerous cell types in the eye constitutively express Fas, the functional outcome of Fas engagement must be constrained, either by cytokines present in the ocular microenvironment, such as TGFβ [51], and/or by some other mechanism. Based on our characterization of the ΔCS mFasL mice, we propose that cleavage of the membrane form of FasL is a critical factor in the regulation of ocular FasL activity in the immune privileged environment of the eye.

While it is well established that FasL is constitutively expressed in the eye, little is known about the extent of cleavage. Cell-free FasL has been identified by ELISA in the ocular fluids of the eye [52], [53]. However, the ELISA assay can not distinguish cleaved sFasL from full-length FasL released from the cell in the form of microvesicles [22]. These studies have been further confounded by irrelevant cross-reactivities of many of the commercially available FasL-specific antibodies. In the current study we rigorously compared whole cell lysates from wild type, ΔCS, and FasL KO mice for FasL expression using a highly specific anti-peptide rabbit antiserum for Western blot analysis [24]. The ΔCS mFasL mice express a gene-targeted form of FasL in which the major cleavage sites were mutated to render the molecule resistant to metalloproteinases. While we were able to distinguish clearly two bands corresponding to full-length FasL in lysates from ΔCS mice, we could barely detect a comparable band in lysates derived from wild type mice. Thus it appears that most ocular FasL is cleaved under normal physiological conditions. Our inability to detect sFasL in the eye lysates from WT mice may reflect increased clearance or instability of the soluble form.

There is general agreement that RGCs die via apoptosis in glaucoma, although the molecular events underlying RGC loss are still debated. The participation of glial cells in the death of RGCs was demonstrated in work from Nakazawa and colleagues linking increased intraocular pressure with a rapid upregulation of retina-associated TNFα and subsequent activation of microglia in the optic nerve head [7]. While both TNFα and activated microglia were required for the death of RGCs, the direct cytotoxic effector mechanism remained unclear. TNFα is detected by 2 receptors; engagement of TNFR1 is thought to trigger apoptosis and engagement of TNFR2 is thought to trigger the Akt signaling cascade and promote survival. TNFR2-deficient, but not TNFR1-deficient mice fail to exhibit RGCs loss following experimentally induced elevated intraocular pressure or TNFα injection, consistent with the premise that TNFα indirectly promotes RGC death [7]. A potential role for FasL, another TNF family member, in glaucoma was suggested by immunohistological examination of the retina in a rat model of glaucoma indicating an increased expression of FasL on microglia in the glaucomatous retina [17].

In the current study we clearly demonstrate that FasL-deficient mice are resistant to TNFα-triggered loss of RGCs subsequent to intravitreal injection. Moreover, TNFα treated ΔCS mice displayed a more rapid loss of retinal ganglion cells and nerve fibers (1 week in ΔCS mice versus 4 weeks in wild type mice). Our observations were not limited to TNFα treated mice. Using the spontaneous DBA/2J model of glaucoma [35], we again observed a significant increase and acceleration in the loss of RGCs and nerve fibers in mice expressing the ΔCS mutation. Unexpectedly, we also observed a dramatic degeneration in all layers of the retina in 12 month old DBA/2J-ΔCS mice. While it is generally accepted that retinal damage in glaucoma patients is restricted to the ganglion cells, there are reports of damage to other types of retinal cells [54]. Taken together, these studies establish the membrane-bound form of FasL as a key neurotoxic effector molecule in glaucoma and suggest that cleavage of FasL is important in protecting retinal tissue from extensive degeneration.

Based on our immunohistological examination of the retina, we further concluded that FasL expression in the retina was predominantly associated with microglial cells and not astrocytes. Prior studies had demonstrated that brain microglia express FasL [55], and therefore it was not surprising to find that retinal microglial cells also expressed FasL. In addition, the neurotoxic effects of FasL in the brain had been previously described in a model of chronic idiopathic demyelinating polyneuropathy, where macrophage-mediated demyelination was shown to be dependent upon FasL mediated death of Schwann cells [56]. Together these data support the hypothesis that RGC-neurotoxic FasL is expressed by retinal microglia. Whether the increased expression of FasL in the retina of TNFα treated mice reflects increased migration of FasL+ microglial cells to the retina and/or increased level of FasL expression/cell remains to be determined. Letellier et al recently demonstrated that FasL triggers migration of Fas+ macrophages and neutrophils into the site of spinal cord injury via activation of the Syk kinase [33]. Importantly, the increased migration triggered via FasL resulted in increased inflammation and tissue damage at the injury site, indicating that FasL triggered migration is a new mechanism by which FasL can trigger destructive inflammation. Whether FasL triggered migration is due to the membrane and/or soluble form of FasL is unknown.

Our data also have important implications for the role of sFasL in glaucoma. The most straightforward explanation for the accelerated loss of RGCs in ΔCS versus WT mice is that greater expression of membrane-bound FasL causes more extensive RGC death. However, we also found that sFasL could antagonize the activity of mFasL and, in the context of glaucoma, be neuroprotective. The opposing activities of membrane and soluble FasL further suggest that FasL cleavage is a major mechanism for limiting the neurotoxic activity of FasL in the eye and raises the intriguing possibility that TNFα may in fact regulate FasL cleavage. The cleavage of FasL is mediated primarily by MMPs (MMP7 and MMP3) as well as TIMPs that are the major endogenous regulators of MMP activities [57]. Both MMPs and TIMPs are expressed by retinal microglia, RGCs, and their axons [58]. Therefore, changes in MMP and/or TIMP expression triggered by TNFα or other factors induced by elevated IOP may be critical in regulating the ratio of soluble to membrane FasL expressed by microglia during glaucoma. In conclusion, our data indicate the enhancement of FasL cleavage and/or forced expression of sFasL may have therapeutic applications in preventing RGC apoptosis in glaucoma.

## Materials and Methods

### Ethics Statement

All animals were treated according to the Association for Research in Vision and Ophthalmology Resolution on the Use of Animals in Research. The Schepens IACUC committee approved all procedures under protocols #S223-1211 and # S230-0312.

### Animals

To examine the importance of sFasL in ocular homeostasis and RGC degeneration, we constructed a FasL-deficient mouse line (designated as FasL KO) and a membrane-only FasL gene-targeted mouse line (this cleavage site deleted-mouse line was designated as ΔCS). The details of how these mice were produced are described in the supplemental data (Figure S1). The ΔCS founder mice were crossed to C57BL/6 mice for one generation and then intercrossed for the TNFα studies. Wild-type littermates were used as WT controls. A second group of mice were backcrossed to DBA/2J mice (Jackson Laboratories), and then intercrossed for in vivo analysis. Assessment of the ΔCS×DBA/2J and wild-type DBA/2J littermates was performed at the fifth generation backcross. FasL knockout mice were described previously [27].

### Intravitreal injections

The intravitreal injection, just posterior to the limbus-parallel conjunctival vessels, were described previously. Mice received a 0.5 µl intravitreal injection of TNF-α (Millipore/Chemicon) (1 ng/0.5 µL of sterile saline) or saline alone. Some mice received recombinant murine soluble FasL alone (R & D Systems) (100 ng/0.5 µl sterile physiological saline) or in combination with the TNFα (100 ng sFasL/1 ng TNFα/0.5 µl sterile physiological saline). The mouse recombinant sFasL corresponds with the physiological cleavage product (Pro 132 to Leu 279) and has weak or no cytolytic activity against A20 target cells.

### Quantitation of retinal ganglion cells

Enucleated eyes were fixed and cryostat sections (8 µm) sections were blocked in 2.5% BSA/0.3% triton in PBS, followed by incubation with a RGC specific primary antibody, anti-βIII-tubulin (TU-20, Millipore), a biotinylated secondary antibody, and Cy2-conjugated streptavidin (Jackson Immuno research). Nuclei were counterstained with To-pro-3 (Molecular Probes). The total number of β-III-tubulin/To-Pro-3 double positive retinal ganglion cells were counted throughout the entire RGC layer of each section. A total of 10 sections were analyzed per eye (10 eyes analyzed per group). The sections were taken through the central globe of each eye.

### Retinal whole mounts

Neural retina was isolated, fixed, and incubated with an anti-neurofilament antibody (SMi32, Covance) for 3 days at 4°C followed by a Rhodamine (TRITC)-conjugated secondary antibody for 2 days at 4°C. Nuclei were counter stained with To-Pro-3 (Molecular Probes, Eugene, OR). Following staining, the retinas were mounted RGC layer side up and examined by confocal microscopy (Leica Microsystems; Wetzlar, Germany).

### Western blots

Protein lysates were prepared from whole eyes (excluding the lens) and splenic T cells stimulated with plate-bound anti-CD3. Proteins were separated on 12% Tris-glycine gels (Invitrogen, Carlsbad, CA), and transferred onto polyvinylidene difluoride membranes (Invitrogen, Carlsbad, CA). The membranes were probed for Fas ligand using a polyclonal rabbit anti-Fas ligand antibody [24] followed by a goat anti-rabbit-HRP secondary antibody (Santa Cruz Biotechnology, Santa Cruz, CA). L5178Y-R tumor transfectants served as positive controls for membrane and soluble FasL.

### Immunofluorescent staining

Immunofluorescent staining was performed on frozen retinal cross sections using primary antibodies to astrocytes (Cy3-conjugated GFAP, Jackson ImmunoResearch), microglia (Iba1, Santa Cruz Technology Inc), FasL (C178 and N20, Santa Cruz technology Inc), and Fas receptor (C20 Santa Cruz technology Inc). The secondary antibody for Iba1, FasL, and Fas was a Cy3-conjugated anti-rabbit. In all cases, isotyped matched antibodies served as negative controls and To-Pro-3 (Molecular Probes, Eugene, OR) was used to stain all nucleated cells.

#### SD-OCT (Spectral Domain Optical Coherence Tomography)

Optical coherence tomography was performed using a SD-OCT system (Bioptigen Inc., Durham, NC) at day 7 after intravitreal TNFα injection. A volume analysis was performed, using 100 horizontal, raster, and consecutive B-scan lines, each one composed by 1200 A-scans. The volume size was 1.6×1.6 cm. The software was able to generate the en-face fundus image using the reflectance information obtained from the OCT sections (volume intensity projection), so that the point-to-point correlation between OCT and fundus position was possible and accurate.

#### In vivo quantification of retinal nerve fiber layer thickness

Sectorial areas containing retinal defects were identified in the retinal whole mount images and the corresponding retinal areas were identified in the OCT-based en-face fundus reconstruction. This was achieved by aligning the two images using the retinal vessels shape and position. Within the OCT images passing through these areas, six sections from each eye were randomly chosen and used by a masked operator to assess the retinal nerve fiber layer thickness. For the measurements, the caliper tool provided by the Bioptigen software was used in 2 different, and randomly chosen positions on the same image. The retinal nerve fiber layer thickness was defined as the interval between the inner and outer boundary of the most internal retinal hyper-reflective layer visualized in the OCT image. Measurement was avoided in OCT points that corresponded to retinal vessels.

### RGC apoptosis

Apoptotic cells were identified in 8 µm frozen retinal sections using a TUNEL In Situ Cell Death detection Kit (TMR red, Roche Applied Science) and sections were mounted using DAPI pan-nuclear stain (Vectashield). Following staining, the ratio of TUNEL positive to total DAPI positive cells was calculated in 6 visual fields at 100× magnification. These calculations were repeated in 3 sections per experimental eye with at least 5 animals per group per time point.

### Intraocular (IOP) measurements

IOP was measured using a TonoLab tonometer (Colonial Medical Supply, Espoo, Finland) and performed as previously described [34].

### Statistics

Where normally distributed, the data were analyzed with an unpaired *t* test with a *P* value of <0.05 as the basis for rejection of the null hypothesis. Statistical analysis and graphing were performed using Microsoft Excel.

## Supporting Information

Figure S1 **Production of the mutant mice.** (A) Targeting vectors designed to delete the 2 cleavage sites (AA 124/125 and AA 127/128) located in exon 2 [59], [60] were constructed from a 129/OLA P1 genomic clone (Genomesystems Inc, St. Louis) and used to transfect 129 ES cells. Appropriately targeted cells were subsequently transfected with the pMC-Cre expression vector to remove the neo cassette [62]. (B) In the Cre-FasL construct, 8 residues (121–128) were deleted from exon 2. This mutation resulted in a splicing error and frameshift mutation, thereby creating a FasL-deficient strain, referred to in the text as FasL KO. By contrast, the ΔCS construct replaced the 4 residues that bracket the 2 potential cleavage sites (designated by the asterisks in the 4 black boxes). These exchange mutations (124_Ser→Thr_, 125_PHe→Leu_, 127_Lys→Arg_, and 128_Gln→Asn_) eliminated the cleavage sites within the full-length protein and prevented the cleavage of FasL to produce the soluble form of FasL. (C) RNA was isolated from activated CD8+ T cells from FasL KO, heterozygous, and wild-type mice. RT-PCR was performed using primers (designated by arrows) to amplify the region spanning exon 1, exon 2, and exon 3. The results demonstrated that wild-type mice expressed a 473 bp fragment indicating all 3 exons are present, while knockout mice expressed only a 427 bp fragment indicating the loss of exon 2. As expected, the heterozygous mice expressed both products. (D) CD8+ T cells were isolated from the spleen and lymph node of: WT, FasL KO heterozygous, and FasL KO homozygous mice. T cells were activated with anti-CD3, and analyzed for FasL expression by FACS analysis. Activated CD8+ T cells from wild-type mice displayed significant levels of FasL, while heterozygous mice displayed a significant reduction in FasL. Activated CD8+ T cells from FasL KO mice displayed no detectable staining over the isotype control antibody, indicating no FasL was expressed on these cells. Similar data was obtained with activated CD4+ T cells (data not shown). (E) Phenotypically, the FasL knock-out mice present with significantly greater splenomegally and lymphadenopathy than *gld*/*gld* mice.
(TIF)

Figure S2 **Apoptosis of RGCs is induced by mFasL in vitro.** RGC5 cells were differentiated in a 96 well microplates (1.5×10^3^ cells per well) as previously described [61]. After differentiation the media was removed and complete DMEM was added. Control vesicles or mFasL vesicles prepared as previously described [13] were added at various dilutions to differentiated RGC5 cells and at incubated at 37°C for 16 hours. Cell viability was assessed using the standard 3-[4,5-dimethylthiazol-2-yl]-2,5-diphenyltetrazolium (MTT) reduction assay. (A) The immortalized RGC-5 cell line was differentiated in vitro and treated with microvesicles expressing membrane-only FasL (mFasL) or no FasL (neo) at increasing concentrations 1∶100, 1∶20, 1∶7. The MTT cytotoxicity assay was used to measure viability and revealed significant loss of viability only in RGCs incubated with mFasL microvesicles. * p>0.05 and ** p>0.01 as compared to media alone.
(TIF)
