# Supplementary figures and images for: Opposing Roles for Membrane Bound and Soluble Fas Ligand in Glaucoma-Associated Retinal Ganglion Cell Death
Source: PLoS One. 2011 Mar 29;6(3):e17659. doi: 10.1371/journal.pone.0017659 (PMC3066205; doi:10.1371/journal.pone.0017659)

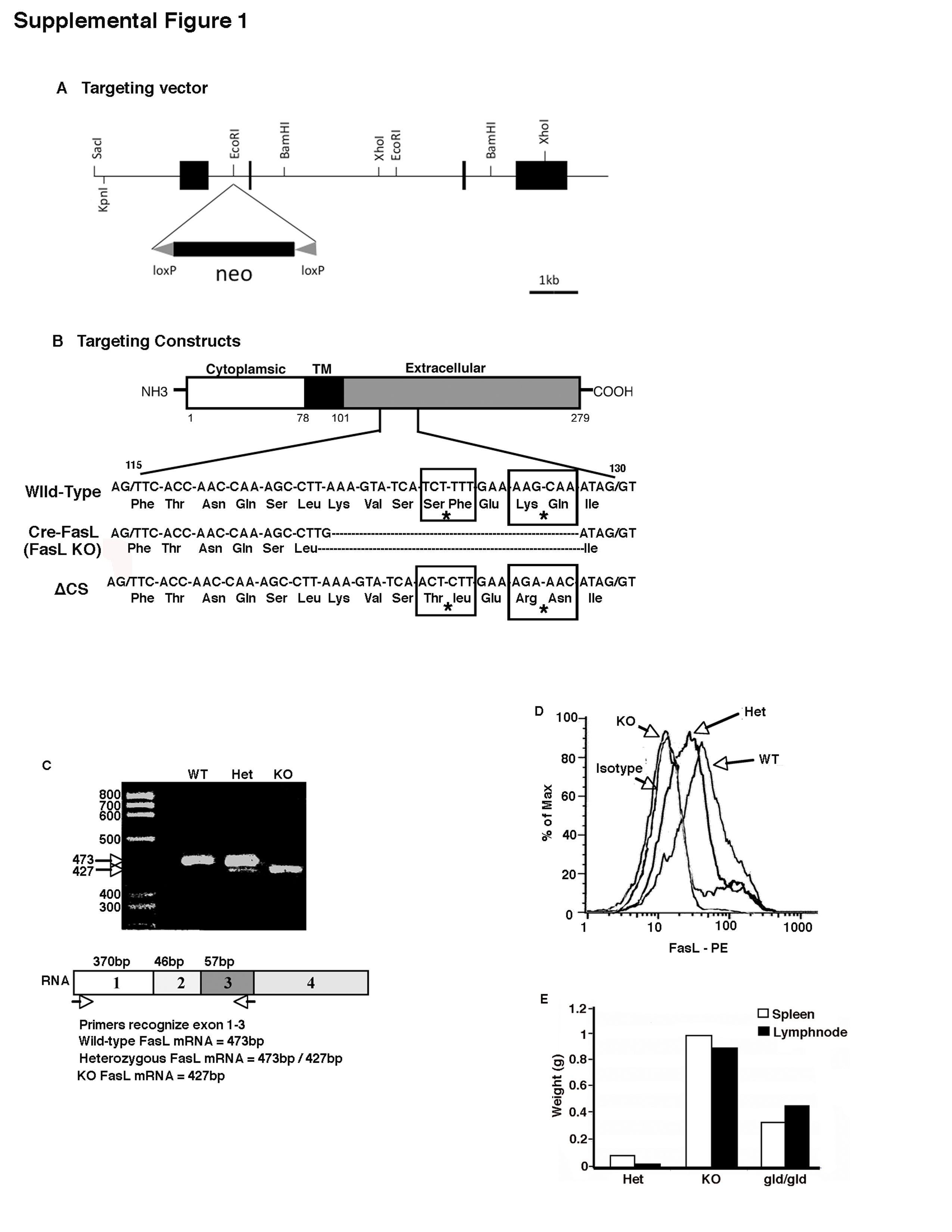

Supplement: Figure S1 — Production of the mutant mice. (A) Targeting vectors designed to delete the 2 cleavage sites (AA 124/125 and AA 127/128) located in exon 2 [59], [60] were constructed from a 129/OLA P1 genomic clone (Genomesystems Inc, St. Louis) and used to transfect 129 ES cells. Appropriately targeted cells were subsequently transfected with the pMC-Cre expression vector to remove the neo cassette [62]. (B) In the Cre-FasL construct, 8 residues (121–128) were deleted from exon 2. This mutation resulted in a splicing error and frameshift mutation, thereby creating a FasL-deficient strain, referred to in the text as FasL KO. By contrast, the ΔCS construct replaced the 4 residues that bracket the 2 potential cleavage sites (designated by the asterisks in the 4 black boxes). These exchange mutations (124Ser→Thr, 125PHe→Leu, 127Lys→Arg, and 128Gln→Asn) eliminated the cleavage sites within the full-length protein and prevented the cleavage of FasL to produce the soluble form of FasL. (C) RNA was isolated from activated CD8+ T cells from FasL KO, heterozygous, and wild-type mice. RT-PCR was performed using primers (designated by arrows) to amplify the region spanning exon 1, exon 2, and exon 3. The results demonstrated that wild-type mice expressed a 473 bp fragment indicating all 3 exons are present, while knockout mice expressed only a 427 bp fragment indicating the loss of exon 2. As expected, the heterozygous mice expressed both products. (D) CD8+ T cells were isolated from the spleen and lymph node of: WT, FasL KO heterozygous, and FasL KO homozygous mice. T cells were activated with anti-CD3, and analyzed for FasL expression by FACS analysis. Activated CD8+ T cells from wild-type mice displayed significant levels of FasL, while heterozygous mice displayed a significant reduction in FasL. Activated CD8+ T cells from FasL KO mice displayed no detectable staining over the isotype control antibody, indicating no FasL was expressed on these cells. Similar data was obtained with activat [file pone.0017659.s001.tif]

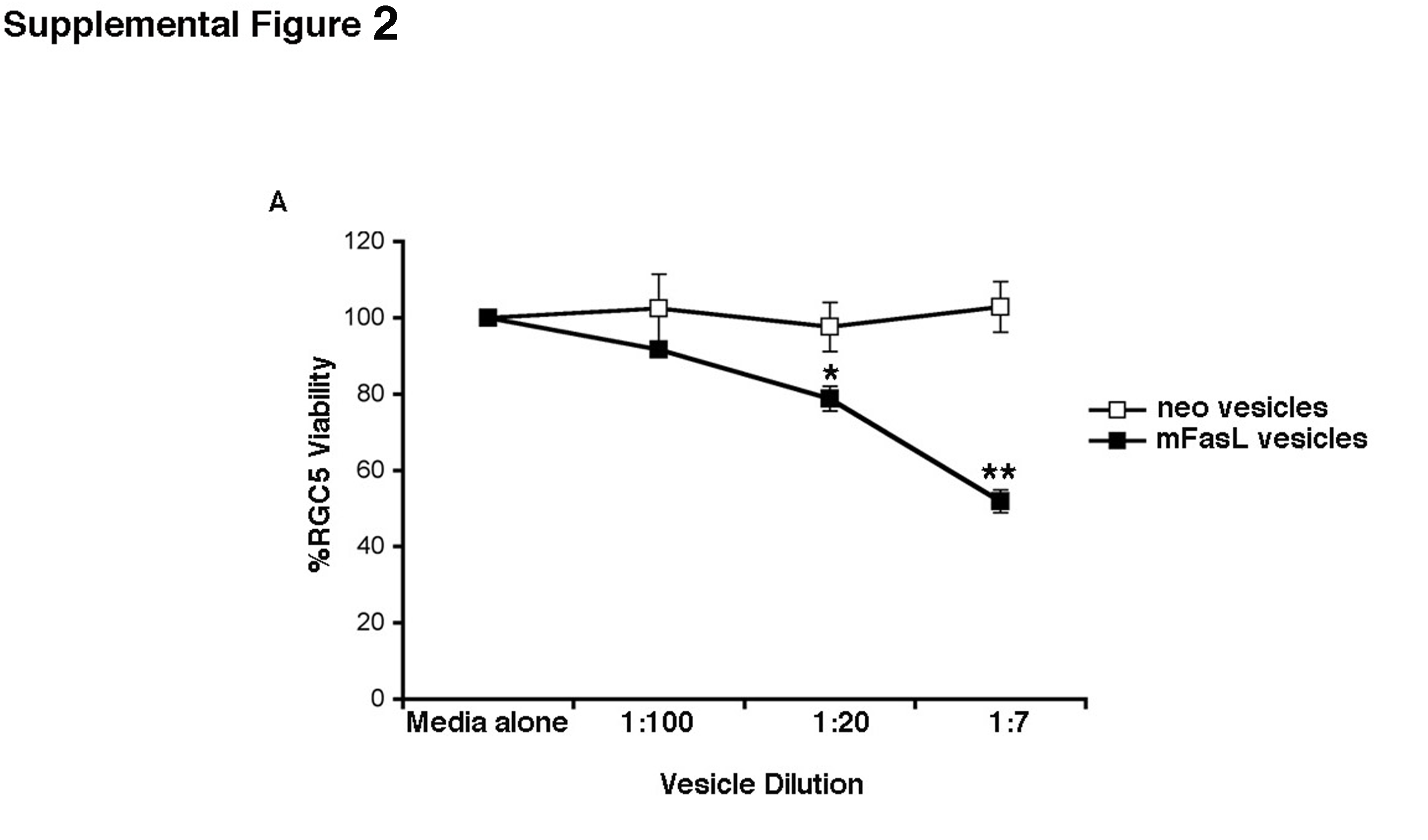

Supplement: Figure S2 — Apoptosis of RGCs is induced by mFasL in vitro. RGC5 cells were differentiated in a 96 well microplates (1.5×10^3^ cells per well) as previously described [61]. After differentiation the media was removed and complete DMEM was added. Control vesicles or mFasL vesicles prepared as previously described [13] were added at various dilutions to differentiated RGC5 cells and at incubated at 37°C for 16 hours. Cell viability was assessed using the standard 3-[4,5-dimethylthiazol-2-yl]-2,5-diphenyltetrazolium (MTT) reduction assay. (A) The immortalized RGC-5 cell line was differentiated in vitro and treated with microvesicles expressing membrane-only FasL (mFasL) or no FasL (neo) at increasing concentrations 1∶100, 1∶20, 1∶7. The MTT cytotoxicity assay was used to measure viability and revealed significant loss of viability only in RGCs incubated with mFasL microvesicles. * p>0.05 and ** p>0.01 as compared to media alone. (TIF) [file pone.0017659.s002.tif]
